# INTERNATIONAL COMPARISON OF GENDER DIFFERENCES IN INFORMANT REPORTS OF COGNITIVE DECLINE IN OLDER ADULTS

**DOI:** 10.1093/geroni/igae098.1153

**Published:** 2024-12-31

**Authors:** Phillip Cantu

**Affiliations:** University of Texas Medical Branch, Galveston, Texas, United States

## Abstract

Informant assessments aid in diagnosing cognitive decline and dementia, yet cultural and social factors may influence reporting. This study investigates gender disparities in informant reports of cognitive function across the US and Mexico using data from Harmonized Cognitive Assessment Protocol (HCAP) of the Health and Retirement Study (HRS) and the Mexican Cognitive Aging Study (MexCOG). Analyzing 4,716 participants, we utilize comparable informant questions and contextual data, including age, gender, education, and MMSE scores. Logistic regressions model the relationship between gender and informant reports separately for each nation. Preliminary analysis reveals gender differences in informant reports of cognitive functioning in both countries. In general, informants were more likely to report symptoms of cognitive decline in the U.S. than in Mexico. Informants were more likely to report memory issues (forgets where things are kept, forgets where they put things), and difficulty with chores for women in both countries. There are fewer gender differences in orientation questions for each nation, maybe related to high level of impairment. In Mexico, informants are more likely to report conversation impairments for women. This study sheds light on the nuanced interplay of cultural and social factors in informant assessments of cognitive function among older adults in diverse national contexts.

